# Exploring Barriers to Compassionate Acts in Individuals with Borderline Personality Disorder: A Critical Literature Review

**DOI:** 10.1002/pmh.70020

**Published:** 2025-04-17

**Authors:** Catrin Street‐Mattox, Matthew R. Broome, Sucharita Maji, Fiona Ng, Lowri Griffiths, Gerald Jordan

**Affiliations:** ^1^ Institute for Mental Health, School of Psychology University of Birmingham UK; ^2^ Birmingham Women's and Children's NHS Foundation Trust UK; ^3^ Department of Humanities and Social Sciences Indian Institute of Technology Dhanbad India; ^4^ Institute of Mental Health, School of Health Sciences University of Nottingham UK; ^5^ Centre for Urban Wellbeing University of Birmingham UK

**Keywords:** adverse childhood experiences, borderline personality disorder, compassionate acts, recovery, stigma

## Abstract

This critical literature review explores the barriers that individuals with borderline personality disorder face when engaging in compassionate acts, including self‐compassion, receiving compassion from others and expressing compassion towards others. Borderline personality disorder is characterised by emotional instability, impulsive behaviours and difficulties in maintaining stable relationships. Although compassionate acts are known to enhance recovery and well‐being, individuals with borderline personality disorder often struggle with these behaviours. This review identifies several key barriers, including adverse childhood experiences, stigma and systemic discrimination, known as sanism, and internal challenges such as self‐judgement, shame and fear of compassion. By synthesising findings from 29 studies, this review highlights the complex interplay between these factors and their impact on the ability of individuals with borderline personality disorder to engage in compassionate behaviours. The findings emphasise the need for personalised, trauma‐informed therapeutic interventions and broader societal changes to foster a more compassionate environment for individuals with borderline personality disorder. Future research should focus on longitudinal studies, inclusion of individuals with lived experiences and exploring diverse sources of compassion to enhance understanding and support recovery in this population.

## 1 Introduction

Borderline personality disorder (BPD) (American Psychiatric Association 2022), also known across some UK NHS boards and in private settings (CAMHS Dumfries and Galloway 2025; PROMIS 2025; The Priory 2022), due to its classification in the ICD‐10 until 2022 (World Health 2004), as emotionally unstable personality disorder (EUPD), personality disorder with borderline pattern (World Health Organisation 2024), or complex emotional needs (Porter et al. 2025), is a multifaceted mental health condition marked by significant emotional instability, impulsive behaviours and challenges in maintaining stable interpersonal relationships (American Psychiatric Association 2022). Individuals with BPD often struggle with regulating their emotions and forming consistent relationships, which can lead to substantial disruptions in various aspects of their lives (World Health Organisation 2024).

Contemporary dimensional models of personality pathology, such as the Alternative Model for Personality Disorders (AMPD) in the Diagnostic and Statistical Manual of Mental Disorders 5th Edition (DSM‐5) and the ICD‐11 model, emphasise that personality disorders are best understood as impairments in self‐ and interpersonal functioning rather than categorical symptom clusters (Sharp et al. 2015). Given that BPD is widely considered a prototypical personality disorder within these frameworks, its defining features, including interpersonal instability and difficulty with trust, highlight the necessity of targeting interpersonal functioning in treatment approaches. Internal barriers to self‐compassion, such as self‐judgment, shame and fear of compassion, can be conceptualised as stemming from broader self‐dysfunction, which in turn perpetuates maladaptive relational patterns. By addressing these barriers, interventions that encourage compassionate acts, whether self‐directed or towards others, may help mitigate core interpersonal difficulties associated with BPD, ultimately supporting recovery.

BPD is prevalent in approximately 1.6% of the general population, yet it accounts for between 10% and 30% of outpatient mental health clinic visits, 20% of psychiatric inpatient admissions and up to 15% of individuals seen in emergency departments for psychiatric reasons (Ellison et al. 2018; National Institute for Health and Clinical Excellence 2009). These statistics highlight the disproportionate burden of BPD on healthcare services compared with other mental health conditions, underscoring the urgent need for targeted therapeutic interventions. Moreover, individuals with BPD are at a high risk of self‐harm and suicidal behaviours, with an estimated 75% engaging in self‐injury and a suicide rate approximately 50 times higher than the general population (Reichl and Kaess 2021). This further emphasises the critical nature of addressing specific therapeutic needs within this population.

Compassionate acts, which encompass a range of prosocial behaviours such as empathy, kindness and altruism towards oneself or others, play an important role in mental health recovery (Flett 2018). These acts can be directed in various ways: compassion from others, compassion towards others and self‐compassion. Engaging in such acts has been shown to enhance a sense of purpose, contribute to recovery and improve overall quality of life for individuals with mental health conditions (Flett 2018; Spandler and Stickley 2011). For individuals with BPD, fostering self‐compassion may be particularly beneficial due to its potential role in mitigating self‐dysfunction, a core feature of personality pathology (Sharp et al. 2015). One of the strongest longitudinal predictors of suicidal behaviour in BPD is identity disturbance, suggesting that interventions targeting self‐perception and emotional regulation may be particularly relevant for reducing distress and risk (Yen et al. 2021). Self‐compassion has been linked to lower levels of shame, self‐judgement and emotional dysregulation, factors that contribute to interpersonal instability (Donald et al. 2019; Naismith et al. 2019). Given that BPD is characterised by difficulties in both self‐ and interpersonal functioning, increasing self‐compassion and compassionate acts towards others may play a crucial role in helping individuals build more stable relationships and regulate distress more effectively.

A focus on compassionate engagement is also particularly relevant given the high levels of social isolation, fear of rejection and self‐stigmatisation associated with the disorder (Klein et al. 2022). Despite possessing the capacity for compassion (Flury et al. 2008), individuals with BPD may experience significant internal and external barriers to acting on compassionate impulses, limiting both their ability to provide compassion to others and their openness to receiving it (Banjeree and Hammond 2022). Moreover, given that BPD is associated with heightened emotional sensitivity and rejection fears, the experience of social stigma may further inhibit compassionate engagement, reinforcing negative self‐perceptions and distress (Corrigan and Watson 2002; Liamputtong and Rice 2021).

This review specifically focuses on compassionate acts rather than compassion motives to examine the behavioural manifestation of compassion. While individuals with BPD may experience compassion‐related emotions, engaging in compassionate acts requires overcoming internal fears, social barriers and maladaptive coping strategies, making it a distinct area of study (Andersson et al. 2022; Jordan et al. 2022a; Jordan et al. 2022b). Compassionate acts also provide tangible benefits for both the recipient and the individual engaging in the behaviour, improving mood, reducing distress and promoting social connection (American Psychological Association 2015; Flett 2018). Given the established role of compassionate acts in mental health recovery (Einat 2017; Jordan et al. 2022a; Jordan et al. 2022b), investigating the barriers that prevent individuals with BPD from engaging in these acts is crucial for informing effective interventions and improving outcomes.

While existing research highlights the benefits of compassionate acts for individuals with BPD and mental health conditions more broadly, the specific barriers that individuals with BPD face in engaging in these acts remain underexplored. Most studies have concentrated on the challenges clinicians encounter in expressing compassion towards individuals with BPD, rather than investigating how individuals with BPD can be empowered to participate in compassionate behaviours themselves.

Recent mental health literature has increasingly focused on identifying the negative impacts of some social experiences on mental health outcomes, recognising that these factors significantly impact recovery processes (Barry et al. 2022; Brandt et al. 2022; Tew et al. 2012; Tzouvara et al. 2023). In this context, stigma and social exclusion are particularly relevant, as they can profoundly influence the ability of individuals with BPD to engage in compassionate acts. Goffman's (1963) conceptualisation of stigma refers to an attribute that discredits an individual, leading to their devaluation and marginalisation by society. This devaluation can manifest as discrimination, stereotyping and social distancing, which are known to have detrimental effects on mental health (Link and Phelan 2001; Rüsch et al. 2005). For example, stigma may result in social withdrawal, thereby limiting opportunities for prosocial engagement, while social isolation might reduce an individual's capacity to experience and express compassion toward others. Minority stress theory further emphasises that individuals from stigmatised or marginalised groups, such as those with mental health disorders, experience chronic stress due to societal prejudice and discrimination, which exacerbates mental health challenges and impedes recovery (Frost and Meyer 2023; Meyer 2003). This interplay between stigma, social exclusion and mental health is critical for understanding barriers to compassionate behaviours in individuals with BPD.

The lack of a comprehensive conceptual understanding of the barriers to compassionate acts among individuals with BPD represents a significant knowledge gap. Without a synthesis of evidence on these barriers, there is a risk that care practices may fall short of supporting recovery in a holistic and effective manner. To address this gap, it is necessary to consolidate diverse sources of evidence to develop a conceptual framework that outlines the barriers to compassionate acts among individuals with BPD.

To establish a foundational understanding of this topic, this critical literature review will draw on and evaluate existing research relevant to the topic to identify and analyse these barriers. By synthesising insights from current literature, this review aims to propose hypotheses and offer recommendations for future research. This approach will contribute to a deeper understanding of the systemic and individual factors that hinder compassionate engagement among individuals with BPD, ultimately informing more effective, recovery‐oriented care practices.

## 2 Methods

Given the limited number of studies specifically addressing this topic, a systematic review was deemed unsuitable. Instead, a critical review was conducted to explore the barriers to compassionate acts among individuals with BPD as discussed in the existing literature. Critical reviews are particularly valuable in fields where existing information is sparse, as they allow researchers to critique the body of literature while establishing a foundation for future research (Grant and Booth 2009).

Moreover, critical reviews enable researchers to reach beyond a single field, synthesising diverse types of evidence from various disciplines. This approach facilitates the development of comprehensive and nuanced conceptual frameworks, drawing on insights from multiple sources to enrich understanding (Paré et al. 2015). By integrating perspectives from different domains, researchers can generate innovative hypotheses and models that are essential for advancing knowledge in complex areas like BPD.

**FIGURE 1:**
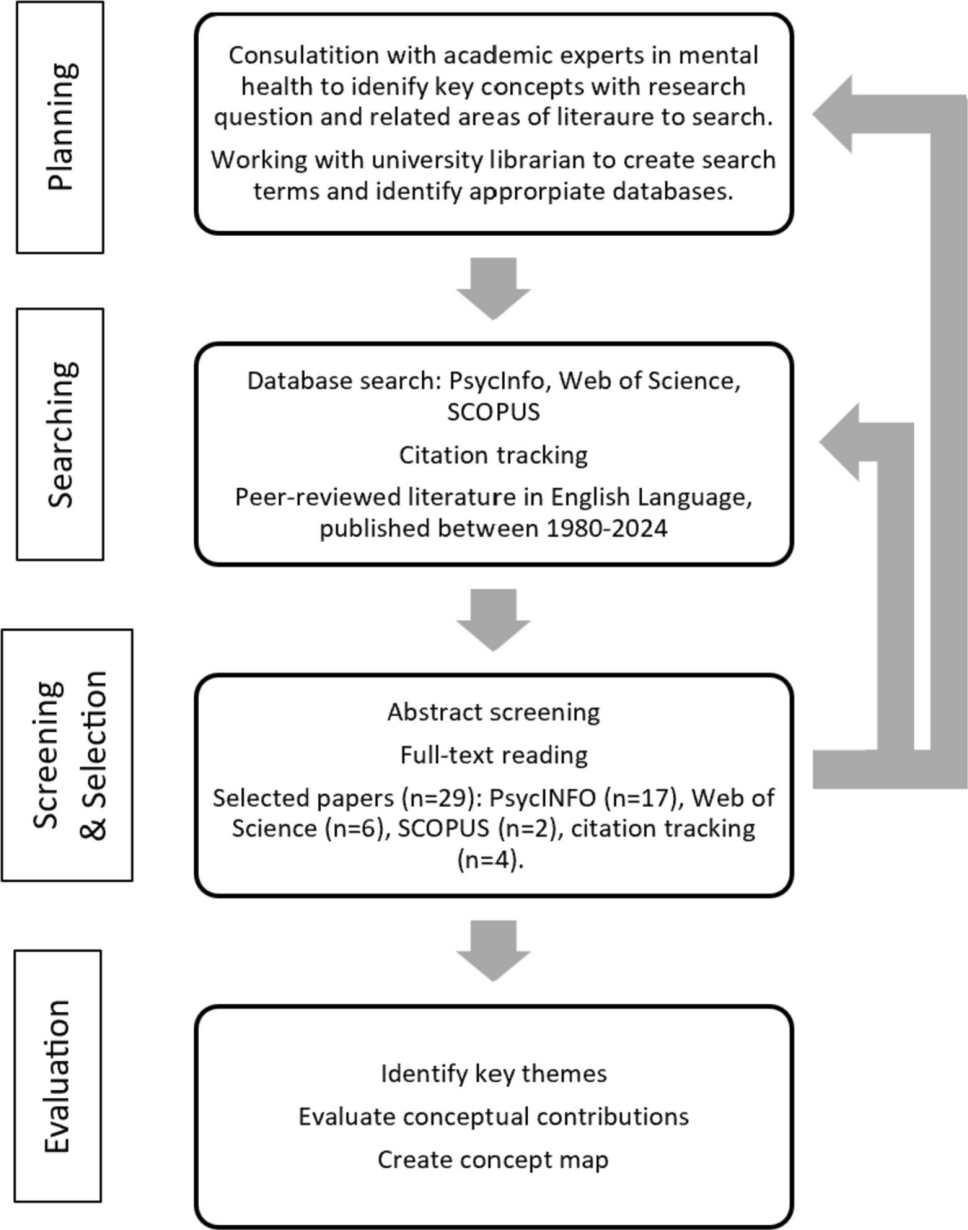
Process diagram outlining the stages of a critical literature review exploring barriers to compassionate acts in individuals with borderline personality disorder. The review followed four stages: planning, searching, screening and selection and evaluation. Databases used included PsycINFO, Web of Science and SCOPUS.

This critical review followed an iterative process, which included continuous modifications to literature gathering based on feedback. The review was conducted in four phases: planning, searching, screening and evaluation (Figure 1).

### 2.1 Planning

**FIGURE 2:**
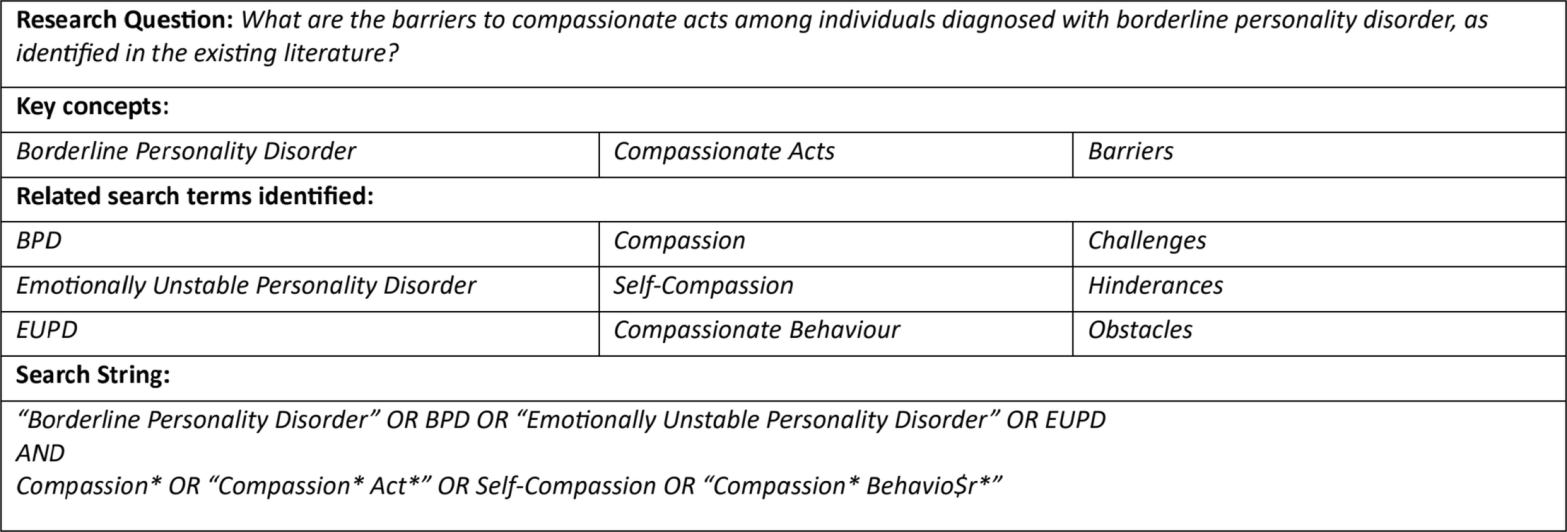
Search strategy table showing key concepts, related search terms, and Boolean search string used to identify relevant literature on compassionate acts and barriers in borderline personality disorder.

During the planning phase, the academic supervisory team specialising in the field of mental health (Prof. Matther Broome, Dr. Sian Lowri Griffiths and Dr. Gerald Jordan), along with Catrin Street‐Mattox, convened to identify key concepts that could be used to answer the research question: ‘What are the barriers to compassionate acts among individuals with borderline personality disorder, as identified in the existing literature?’. The key concepts identified were BPD, compassionate acts and the barriers to these acts. With the help of a university librarian, these were developed into search terms to be used in relevant databases (Figure 2).

### 2.2 Searching

**FIGURE 3:**
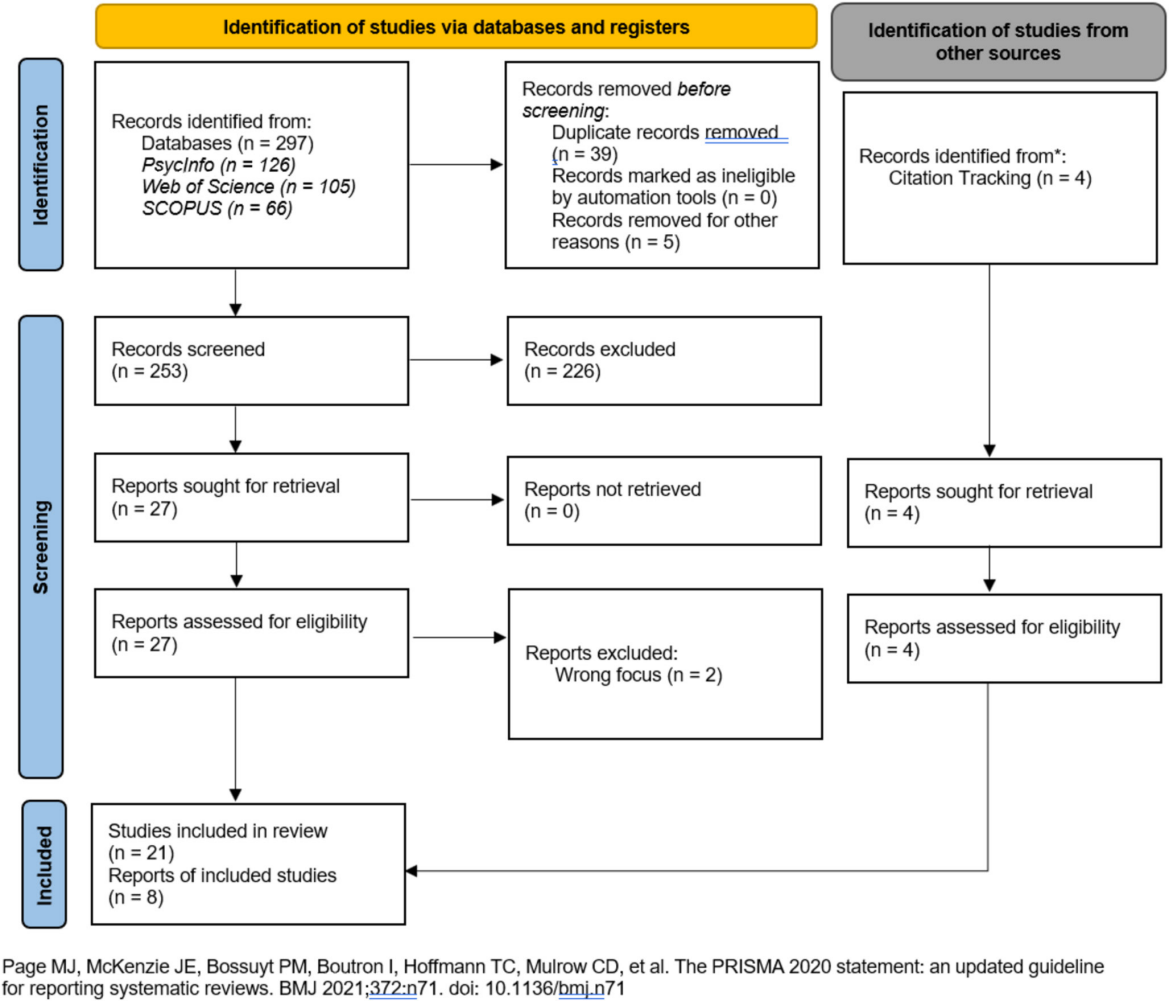
Preferred Reporting Items for Systematic Reviews and Meta‐Analyses (PRISMA) flow diagram outlining the identification, screening, and inclusion process of studies from databases and citation tracking, resulting in 29 sources for final review. The diagram follows PRISMA 2020 guidelines.

Search terms and databases were identified through consensus between the study team, a university librarian, and by examining the number of hits generated through pilot testing of keywords. The final search terms focused on the concepts of BPD and compassionate acts, and the databases selected for the search were PsycINFO, Web of Science and SCOPUS. This search yielded 297 results, and after removing 39 duplicates, 258 papers remained for screening (Figure 3).

Studies were included if they were published in English between 1980, the year in which BPD was added to the DSM‐III (American Psychiatric Association 1980), and 2024, and if they focused on the relationship between BPD and compassionate acts (Oldham 2009). Studies that examined core features of BPD, such as emotional dysregulation, identity disturbance, or fear of abandonment, were included, even if participants were not formally diagnosed. These studies were included to acknowledge that BPD features exist on a continuum, and investigating these traits in non‐clinical samples can provide valuable insights into the barriers to compassionate acts. However, we acknowledge this as a limitation in fully capturing the experiences of those with a formal BPD diagnosis. To ensure transparency, we have documented how BPD was measured in each included study in Table 1, which details whether participants were formally diagnosed or identified based on screening tools.

**TABLE 1:** Summary of literature included in review.

Author(s)	Journal	Type of work	Country and sample info	Aim	BPD measure	Key themes
**PsycINFO**						
(Carreiras et al. 2022)	*European Journal of Developmental Psychology*	PR Journal Article Original Study Quantitative	Portugal Female *n* = 422 Mean age = 15.40 years	Examine the role of self‐compassion‐ focusing on components including mindfulness, isolation, and self‐judgment, and their role in mitigating borderline features in nonclinical adolescents.	BFPFS‐C	Self‐compassion Recovery
(Donald et al. 2019)	*Australasian Psychiatry*	PR Journal Article Original Study Quantitative	Australia Female *n* = 17, not‐female *n* = 2 Average age = 33.47 years	Investigate the association between self‐compassion, self‐criticism, and personal recovery in individuals with BPD.	SCID‐IV‐TR‐BPD	Self‐compassion Recovery
(Fagan 2017)	N/A	Doctoral Thesis Lancaster University Qualitative	UK Female *n* = 6 Mean age = 41.7 years	Challenge the reductionist narrative of BPD by exploring a relational understanding of BPD and integrating the construct of compassion.	Diagnosis confirmed via screening questions	Compassion from others Stigma
(Feliu‐Soler et al. 2017)	*Clinical Psychology & Psychotherapy*	PR Journal Article Quantitative Original Study	Spain Female = 30, Male = 2 Age: 18‐45 years	Investigate the effects of a short training program in loving‐kindness and compassion meditation (LKM/CM) in patients with BPD.	BID‐R	Self‐compassion Recovery
(Jorgensen and Boye 2024)	*Personality Disorders: Theory, Research, & Treatment*	PR Journal Article Qualitative Original Study	Denmark Female *n* = 21 Mean age = 27.27 years	Investigate how severe shame manifests in the subjective experience and behaviour of women diagnosed with BPD.	Clinical recruitment, DSM‐5 criteria	Self‐compassion Recovery
(Krawitz 2012a; Krawitz 2012b)	*Australasian Psychiatry*	PR Journal Article *(Parts 1 & 2)* Conceptual Paper	N/A	Outline difficulties in treating severe chronic self‐loathing in people with BPD and to discuss interventions, particularly focusing on self‐compassion.	N/A	Self‐compassion Recovery
(Naismith et al. 2019)	*Clinical Psychologist*	PR Journal Article Qualitative Original Study	UK Female *n* = 44, Male *n* = 9 Mean age = 32 years	Explore inhibitors and facilitators for clients with personality disorders trialling compassion‐focused imagery (CFI) over 1 week.	Outpatient clinic recruitment, DSM‐5 criteria	Self‐compassion Recovery
(Pohl et al. 2021)	*Journal of Clinical Psychology*	PR Journal Article Quantitative Original Study	Germany Female *n* = 70 *(BPD:n = 35; CG:n = 35)* Mean age = 28.57 years	Investigate whether (1) patients with BPD show lower self‐compassion and self‐esteem versus healthy controls and (2) do these factors moderate the association between childhood trauma and the severity of BPD symptoms?	Clinical recruitment, DSM‐IV criteria	Self‐compassion Recovery
(Rivera 2013)	N/A	Doctoral Thesis Quantitative Alliant International University	USA Female *n* = 28, Male *n* = 12 Age range: 18–65 years	Determine if deficits in mindfulness or self‐compassion are components of BPD symptomatology, and to investigate the relationships between mindfulness, self‐compassion, and BPD characteristics.	BPQ	Self‐compassion Recovery
(Salgó et al. 2021)	*PLOS ONE*	PR Journal Article Quantitative Original Study	Hungary Female *n* = 144, Male *n* = 25 (BPD:*n* = 50, CG: *n* = 70) Mean age = 30.7 years	Investigate emotion regulation difficulties specific to BPD compared to a healthy control group, focusing on mindfulness and self‐compassion as potential factors.	Clinical recruitment	Self‐compassion Recovery
(Scheibner et al. 2018)	*Journal of Personality Disorders*	PR Journal Article Quantitative Original Study	Germany Female *n* = 80, Male *n* = 64 (BPD: *n* = 28, CG:*n* = 116) Age range: 18–59 years	Explore how self‐compassion mediates the relationship between mindfulness and BPD symptoms, specifically focusing on emotion dysregulation.	Structured Clinical Interview for DSM‐IV Axis II Personality Disorders	Self‐compassion Recovery
(Soler et al. 2022)	*BPD and Emotion Dysregulation*	PR Journal Article Qualitative Original Study	Spain Female *n* = 30, Male *n* = 2 Mean age = 40.3 years	Assess the feasibility and acceptability of an 8‐week group intervention for long‐lasting BPD, focussing on perspective of recovery.	Clinical recruitment, DSM‐IV‐TR, DIB‐R	Self‐compassion Recovery
(Sommerfeld and Bitton 2020)	*Frontiers in Psychology*	PR Journal Article Quantitative Original Study	Israel Female *n* = 36, Male *n* = 24 Mean age = 26.8 years	Investigate whether borderline features mediate the associations between rejection sensitivity, self‐compassion, and aggressive behaviour in adults.	Support group recruitment, confirmed clinician diagnosis	Self‐compassion Recovery
(Southward et al. 2023)	*Journal of Affective Disorders*	PR Journal Article Quantitative Original Study	USA In person: Female *n* = 161, Male *n* = 111, Mean age = 36.4 years Online: Female *n* = 90, Mean age = 32.14 years	Examine the associations between BPD features and three potential protective factors: conscientiousness, distress tolerance, self‐compassion.	PAI‐BOR	Self‐compassion Recovery
(Valikhani et al. 2020)	*Current Psychology*	PR Journal Article Quantitative Original Study	Iran Female *n* = 211, Male *n* = 221 Mean age = 24.61 years	Investigate dimensional personality disorder traits in a nonclinical sample through the lens of Three‐Dimensional Model of Personality Self‐Regulation (TDMPS), focusing on self‐control, self‐knowledge/mindfulness, and self‐compassion as core elements of self‐regulation.	PDQ‐4+	Self‐compassion Recovery
(Warren 2015)	*Personality & Mental Health*	PR Journal Article Commentary	N/A	Explore how self‐criticism, shame, and self‐compassion may influence treatment outcomes and therapeutic approaches for people with BPD.	N/A	Self‐compassion Recovery
**Web of Science**						
(Fagan et al. 2022)	*Psychological Reports*	PR Journal Article Qualitative Original Study	UK Female *n* = 6 Age range: 28–57 years	Explore the lived experiences of compassion among individuals diagnosed with BPD. Develop a model of compassion specific to BPD and understand how compassion influences the recovery process.	Diagnosis confirmed via screening questions	Self‐compassion Compassion from others Compassion towards others Recovery
(Gratz et al. 2022)	*BPD and Emotion Dysregulation*	PR Journal Article Quantitative Original Study	USA Female *n* = 37, Male *n* = 56 Mean age = 32.61 years	Investigate how BPD symptoms relate to ineffective conflict resolution strategies in romantic relationships involving substance use, with a particular focus on the roles of fear of compassion from others and for others.	Clinical recruitment	Compassion towards others Compassion from others Recovery
(Katsakou et al. 2019)	*Journal of Mental Health*	PR Journal Article Qualitative Original Study	UK Service users: Female *n* = 39, Male *n* = 9, Mean age = 36.5 years Therapists: Female *n* = 9, Male *n* = 8, Mean age = 40.1 years	Explore how recovery in BPD occurs through routine or specialist treatment, as perceived by service users and therapists.	Clinical recruitment	Compassion from others Self‐compassion Recovery
(Keng and Wong 2017)	*BPD and Emotion Dysregulation*	PR Journal Article Quantitative Original Study	Singapore Female = 209, Male = 81 Mean age = 19.93 years	Investigate the association among childhood invalidation, self‐compassion and BPD symptoms among a non‐clinical sample of university students.	PAI‐BOR	Self‐compassion Recovery
(van Schie et al. 2024)	*Personality & Mental Health*	PR Journal Article Qualitative Original Study	Australia Consumers: Female *n* = 30, Male *n* = 3, Mean age = 34.1 years Carers: Female *n* = 26, Male *n* = 4, Mean age = 42.4 years	To explore the language used regarding BPD and its effect on those with BPD and their carers.	Recruited from support forum	Self‐compassion Compassion from others Recovery
(Wilner et al. 2024)	*Journal of Personality Disorders*	PR Journal Article Conceptual Paper	N/A	Describe the nature of self‐hatred in BPD and propose a theory for its development.	N/A	Self‐compassion Recovery
**SCOPUS**						
(Koivisto et al. 2022)	*European Journal for Qualitative Research in Psychotherapy*	PR Journal Article Mixed Methods Original Study	Finland Female *n* = 7, Male *n* = 1 Mean age = 30 years	Explore how development and change in self‐concept and identity were maintained over a 12‐month follow‐up period in individuals with BPD after attending a psychoeducational intervention.	Clinical recruitment, DSM‐5	Self‐compassion Recovery
(Rajabi et al. 2023)	*Iranian Rehabilitation Journal*	PR Journal Article Quantitative Original Study	Iran Female *n* = 153, Male *n* = 147 Mean age = 21 years	Explore pathology of BPD symptomatology in a nonclinical sample and assess the roles of mental pain, cognitive emotion regulation, self‐compassion, and depression.	PAI‐BOR	Self‐compassion Recovery
**Citation Tracking**						
(Grzegorzewski et al. 2019)	*Psychiatry Research*	PR Journal Article Quantitative Original Study	UK Female *n* = 30 Mean age = 27.3 years	Examine cognitive and affective empathy and altruism in women with BPD and their impacts on clinically relevant factors such as alexithymia, anxiety, and depressive symptoms.	BPD‐5, ICD‐10	Compassion for others Recovery
(Holm and Severinsson 2008)	*International Journal of Mental Health Nursing*	PR Journal Article Literature Review	N/A	Synthesise existing literature on emotional pain and distress in women with BPD, examining the prevalence, interventions, and effectiveness of various treatments.	N/A	Compassion from others
(Moltu et al. 2023)	*International Journal of Qualitative Studies on Health and Well‐being*	PR Journal Article Qualitative Original Study	Norway Female *n* = 12 Age range: 21–27 years	Explore how people with BPD experience relationships with themselves and others.	Diagnosis confirmed, and in treatment	Self‐compassion Compassion from others
(Ociskova et al. 2023)	*Neuroendocrinology Letters*	PR Journal Article Literature Review	N/A	Review current knowledge about stigma and self‐stigma in BPD.	N/A	Self‐compassion Compassion from others Compassion towards others

Sources of evidence were excluded if they primarily focused on mental health conditions other than BPD without substantial relevance to BPD and compassion, or if they did not explore the experiences of or engagement in compassionate acts in relation to BPD. Specifically, studies were excluded if they did not examine how individuals with BPD, or symptoms of BPD, engage in compassionate acts, or their experiences of receiving or showing compassion. For example, papers that discussed why clinicians should be compassionate towards patients with BPD but did not address the perspective or experiences of individuals with BPD regarding receiving or giving compassion were excluded. These exclusion criteria were established to ensure that the review remained focused on the intersection of BPD and compassion, providing the most relevant insights for the development of a conceptual framework.

### 2.3 Screening and Selection

Articles identified from the search were subjected to a two‐step screening process: title and abstract screening followed by full‐text screening. This process was conducted using EndNote by one researcher to ensure consistency. The title and abstract screening focused on identifying studies that explicitly addressed BPD in relation to compassionate acts. Studies that met the initial screening criteria were then subjected to a full‐text review to assess their relevance to the topic. The primary author conducted this phase with support from the wider team when necessary.

### 2.4 Evaluation

During the evaluation phase, each paper selected for full‐text screening was read thoroughly by the primary researcher. Detailed notes were taken on each paper, including the type of study, key themes explored, the forms of compassionate acts investigated, as well as the strengths and limitations of each study. These notes were used to develop a conceptual map of interrelated key concepts surrounding the topic, which would inform the subsequent critical review.

## 3 Results

### 3.1 Characteristics of Included Studies

A total of 258 papers were identified during the search phase. Many did not specifically examine barriers to compassion but instead focused on compassion levels in individuals with BPD. Studies that explored factors influencing compassion levels and the impact of interventions for individuals with BPD were included, while those examining personality disorders more broadly without a specific focus on BPD were excluded. Papers discussing ‘borderline traits’ or ‘borderline symptoms’ rather than a full BPD diagnosis were included if they provided relevant insights.

Additionally, many studies focused on compassion towards individuals with BPD, such as compassion fatigue among carers or challenges in delivering compassionate care, without addressing how individuals with BPD themselves engage in compassion. These were excluded. After applying the inclusion criteria, 29 papers were selected for review (Table 1).

The included papers consisted mostly of peer‐reviewed articles (*n* = 27), with the majority being original empirical studies (*n* = 21). In addition to empirical studies, the review included peer‐reviewed conceptual papers (*n* = 2), a commentary (*n* = 1), literature reviews (*n* = 2) and doctoral theses that included empirical studies (*n* = 2). Empirical and grey literature, such as doctoral theses, were included to ensure a comprehensive understanding of the topic, particularly given the limited research available on BPD and compassionate acts.

The original studies (*n* = 23) included in the review, including both doctoral theses, employed both qualitative (*n* = 9) and quantitative (*n* = 14) methods. One study utilised mixed methods (*n* = 1). Although some studies used Interpretative Phenomenological Analysis (IPA) (*n* = 2) to explore lived experiences, none reported involving individuals with lived experience in the research design or authorship.

Regarding the geographic distribution of the original studies, most were conducted in Western, Educated, Industrialised, Rich, and Democratic (WEIRD) countries (*n* = 20), including the United States, Australia, New Zealand and several countries across Europe and the Middle East, with other study locations including Iran (*n* = 2) and Singapore (*n* = 1).

The demographic characteristics of participants varied across studies, with many studies predominantly including female participants (*n* = 14), and some exclusively focusing on female samples (*n* = 7). Age was reported inconsistently across studies; some provided mean ages, while others reported age ranges. The youngest sample had a mean age of 15.4 years (Carreiras et al. 2022), whereas the oldest participants were reported within an age range of up to 65 years (Rivera 2013). However, most studies focused on young to middle‐aged adults, approximately 18–40 years old.

Several studies in the review (Carreiras et al. 2022; Keng and Wong 2017; Rajabi et al. 2023; Valikhani et al. 2020) examined ‘borderline features’ in ‘healthy participants’ rather than individuals with a formal BPD diagnosis. Only four studies (Fagan 2017; Koivisto et al. 2022; Moltu et al. 2023; van Schie et al. 2024) adopted an approach that went beyond asking set, predefined questions and instead explored participants' broader lived experiences, and instead allowed for exploration of participants' broader lived experiences, allowing them to discuss how BPD fits into their lives as a whole.

In relation to the key concept of compassion, the majority of papers (*n* = 25) investigated compassionate acts in the form of ‘self‐compassion’. Additionally, eight papers explored the experience of receiving compassion from others, while a smaller number (*n* = 3) addressed the experiences of acting compassionately towards others.

### 3.2 Critical Review of Evidence

#### 3.2.1 Compassion as a Tool for BPD Recovery

Studies included in this review (*n* = 29) highlight the significant role of compassion in BPD recovery, demonstrating its impact across three key areas: self‐compassion, receiving compassion from others and expressing compassion toward others. Receiving compassion from mental health professionals is consistently linked to improved therapeutic alliances, reduced stigma and enhanced self‐perception, which in turn contribute to more positive recovery trajectories (Fagan 2017; Fagan et al. 2022; Katsakou et al. 2019; van Schie et al. 2024). The ability to practice self‐compassion has also been shown to support emotional regulation, lower levels of self‐criticism, and reduced symptom severity, reinforcing its potential as a protective factor against the psychological distress characteristic of BPD (Carreiras et al. 2022; Donald et al. 2019; Feliu‐Soler et al. 2017; Grzegorzewski et al. 2019; Jorgensen and Boye 2024; Keng and Wong 2017; Koivisto et al. 2022; Krawitz 2012a, 2012b; Naismith et al. 2019; Pohl et al. 2021; Rivera 2013; Salgó et al. 2021; Scheibner et al. 2018; Sommerfeld and Bitton 2020; Valikhani et al. 2020; Wilner et al. 2024). Meanwhile, difficulties in engaging compassionately with others are associated with relational instability and an increased risk of relapse, particularly in individuals with co‐occurring conditions like addiction (Gratz et al. 2022).

Empirical studies further support the therapeutic potential of compassion‐focused interventions in BPD treatment. Structured programs such as loving‐kindness and compassion meditation (LKM/CM) have been shown to improve self‐compassion and emotional regulation (Feliu‐Soler et al. 2017), while research on self‐compassion in BPD populations suggests that higher levels of self‐compassion are associated with better personal recovery outcomes and reduced symptom severity (Pohl et al. 2021). However, barriers to compassionate acts remain a critical challenge, with evidence indicating that fears of receiving and expressing compassion can contribute to interpersonal conflict, avoidance of care and diminished recovery outcomes (Gratz et al. 2022).

These findings underscore the need to identify and address barriers to compassionate acts, as doing so could enhance therapeutic interventions and optimise recovery outcomes.

#### 3.2.2 Barriers to Compassionate Acts

This review identified three primary barriers to compassionate acts among individuals with BPD: Adverse Childhood Experiences (ACEs), Stigma and Internal Barriers to Self‐Compassion. These barriers, often deeply ingrained, significantly impair individuals' ability to engage in compassion‐based behaviours.

##### 3.2.2.1 Adverse Childhood Experiences (ACEs)

Adverse childhood experiences (ACEs), including abuse, neglect, domestic violence and childhood invalidation, are strongly linked to the development and severity of BPD symptoms (Estric et al. 2022). These experiences contribute to core difficulties associated with BPD, such as emotional dysregulation, self‐criticism, and unstable interpersonal relationships, which in turn impact an individual's ability to engage in compassionate acts, both towards themselves and others (Donald et al. 2019; Fagan et al. 2022; Pohl et al. 2021; Rajabi et al. 2023). While self‐compassion has been identified as a potential protective factor in mitigating emotional dysregulation (Pohl et al. 2021), its effectiveness is not universal, as fears of compassion and deep‐seated self‐judgement may limit its benefits for individuals with a history of trauma (Fagan et al. 2022; Naismith et al. 2019).

While self‐compassion has been identified as a factor that may support emotional regulation in individuals with BPD who have experienced ACEs, its role remains complex and variable. While some evidence suggests that higher self‐compassion is associated with lower symptom severity and better emotional regulation (Pohl et al. 2021), other findings complicate this picture. For instance, (Keng and Wong 2017) found that self‐compassion did not moderate the relationship between childhood invalidation and BPD symptoms in a non‐clinical sample, even though it consistently predicted lower symptom severity overall. These discrepancies indicate that the benefits of self‐compassion interventions may depend on factors such as the specific type of ACE experienced, the severity of symptoms, and whether the individual has received a formal clinical diagnosis. The heterogeneity in responses to self‐compassion interventions underscores the need for tailored therapeutic approaches that account for these contextual differences.

A key challenge for those who have experienced ACEs and live with BPD is navigating both the giving and receiving of compassion, a paradox in which offering compassion to others may come more easily than accepting it themselves (Dinsdale and Crespi 2013). While early trauma often leads to heightened emotional sensitivity, relational instability and difficulties in trust, it may also increase an individual's awareness of suffering in others (Fagan et al. 2022). This can lead to a dissociation between outward compassion and self‐directed kindness, where individuals with BPD may engage in compassionate acts towards others but struggle to extend or receive compassion themselves (Fagan et al. 2022). This reluctance to accept compassion is often underpinned by deeply ingrained beliefs of unworthiness and fears of dependency, which can result in emotional shutdown when confronted with genuine care from others (Holm and Severinsson 2008). Research further supports this self‐compassion paradox, demonstrating that difficulties in receiving compassion may reinforce social withdrawal and interpersonal difficulties. Holm and Severinsson (2008) highlight that intense emotional pain, fear of rejection and deep‐seated mistrust act as barriers to compassionate engagement, making it less likely that individuals with BPD will seek or accept support. This aversion to receiving compassion may also stem from experiences of inconsistent or harmful caregiving during childhood, which can lead to defensive avoidance of perceived intimacy or kindness (Holm and Severinsson 2008; Fagan et al. 2022). Consequently, even when external compassion is available, the emotional distress associated with vulnerability can prevent individuals with BPD from fully benefiting from it.

These internal conflicts not only shape interpersonal relationships but also influence responses to therapeutic interventions that emphasise self‐compassion. While compassion‐focused therapy (CFT) and similar approaches are often recommended to improve emotional regulation and self‐acceptance, research suggests that individuals with a history of ACEs may perceive these interventions as invalidating or even emotionally overwhelming (Donald et al. 2019; Soler et al. 2022). Donald et al. (2019) found that self‐compassion exercises can provoke negative reactions in individuals with BPD, particularly those who experience strong self‐criticism and entrenched shame. This is consistent with findings from Soler et al. (2022), who reported that participants in an eight‐week recovery‐focused group intervention experienced distressing emotions such as grief and shame during self‐compassion exercises, likely tied to unprocessed trauma and feelings of unworthiness. These findings highlight the importance of careful implementation when introducing self‐compassion‐based interventions for individuals with a history of ACEs. Rather than assuming that self‐compassion will be universally beneficial, interventions must be gradual, structured and trauma‐informed to prevent individuals from feeling overwhelmed or alienated by practices intended to foster healing (Donald et al. 2019; Soler et al. 2022). Mindfulness‐based approaches, for example, may help individuals increase awareness of their emotional responses to compassion‐related exercises while also providing strategies to manage distressing reactions (Pohl et al. 2021). However, a staged and individualised approach is crucial to avoid exacerbating self‐stigma and emotional avoidance.

Given the complex relationship between ACEs, self‐compassion and relational dynamics, it is clear that self‐compassion‐focused interventions must be integrated into broader treatment frameworks that address both the psychological and interpersonal consequences of early adversity. Approaches such as Dialectical Behaviour Therapy (DBT) and Mentalisation‐Based Treatment (MBT) provide structured ways to address emotional dysregulation, trust difficulties and self‐judgement, which are key barriers to compassionate engagement (Naismith et al. 2019). These therapies, when infused with compassion‐focused elements, can help individuals gradually develop tolerance for self‐directed kindness, particularly when introduced alongside emotion regulation and distress tolerance skills. Furthermore, a trauma‐informed approach that prioritises safety, validation and gradual exposure to self‐compassion exercises may enhance engagement with compassion‐focused interventions. Research suggests that delivering these interventions within a framework that acknowledges the impact of ACEs on self‐perception and interpersonal trust can help reduce resistance to self‐compassion practices (Soler et al. 2022; Fagan et al. 2022). This could involve progressive exercises that start with less emotionally intense forms of self‐compassion, such as neutral self‐talk or cognitive restructuring, before transitioning into more direct self‐kindness practices.

The relationship between ACEs and barriers to compassionate engagement in BPD is complex and multifaceted. While self‐compassion has been identified as a potential protective factor, fear of compassion, emotional shutdown and internalised shame often limit its effectiveness. Moreover, individuals with BPD may exhibit high levels of outward compassion towards others while simultaneously rejecting or avoiding self‐compassion, reinforcing cycles of distress and social isolation.

##### 3.2.2.2 Stigma

Individuals with BPD often face significant stigma and misunderstandings surrounding their diagnosis, which can contribute to iatrogenic harm, exacerbating symptoms and hindering recovery (Grambal et al. 2016; Klein et al. 2022; Weinbrecht et al. 2018). Stigma, broadly defined as discrimination or prejudice against individuals based on perceived mental illness, can manifest in various ways, including public stigma (negative societal attitudes), self‐stigma (internalised negative beliefs) and structural stigma (discriminatory policies or practices within institutions) (Corrigan and Watson 2002; Thornicroft et al. 2007). These layers of stigma create significant barriers to compassionate engagement, both in clinical settings and personal relationships, as individuals with BPD may anticipate or experience rejection from others.

Within clinical settings, stigma is often reinforced by negative perceptions of BPD. Individuals with BPD may engage in behaviours that clinicians interpret as challenging, such as emotional outbursts, self‐harm, or inconsistent engagement with treatment (Aviram et al. 2006; Bodner et al. 2015). While these behaviours can be understood as expressions of emotional distress and interpersonal difficulties, they are frequently misconstrued as manipulative or attention‐seeking, leading to negative clinician attitudes (Aviram et al. 2006). These attitudes, in turn, can weaken therapeutic alliances, making individuals with BPD less likely to engage in care and more vulnerable to further rejection, thereby reinforcing a cycle of social and clinical exclusion.

Research highlights that stigma has direct consequences for treatment access, therapeutic relationships and self‐perception. Clinicians who hold negative views of individuals with BPD may be less willing to provide supportive and effective treatment (Fagan 2017). This is particularly concerning given that therapeutic alliance is a central predictor of treatment outcomes, and disruptions in trust between individuals with BPD and clinicians can hinder engagement in care (Klein et al. 2022). Furthermore, individuals with BPD are often labelled with pejorative terms such as ‘attention‐seeking’ or ‘manipulative’, reinforcing the notion that they are ‘difficult’ or ‘untreatable’ (Krawitz 2004; Ring and Lawn 2019). Such characterisations may contribute to exclusion from services, inconsistent treatment, or inadequate support, creating additional barriers to recovery (Klein et al. 2022).

The relationship between stigma and self‐compassion has also been explored in the literature, demonstrating how external stigma can become internalised, further reinforcing psychological distress. van Schie et al. (2024) found that stigmatising language used by clinicians can foster feelings of inadequacy and frustration in individuals with BPD, ultimately leading to lower levels of self‐compassion. Similarly, Ociskova et al. (2023) reviewed existing research on stigma and self‐stigma in BPD, noting that individuals often face negative attitudes from both the general public and healthcare professionals, which has been associated with diminished engagement in compassionate acts. The role of stigma in shaping self‐perception, emotional regulation, and recovery trajectories suggests that interventions aimed at reducing stigma could have downstream benefits in fostering self‐compassion and improving mental health outcomes (Koivisto et al. 2022).

##### 3.2.2.3 Internal Barriers to Self‐Compassion

Individuals with BPD face several internal barriers that hinder their ability to engage in self‐compassion. These include deeply ingrained self‐judgment and self‐criticism, pervasive feelings of shame and unworthiness, difficulties with cognitive empathy, and a pronounced fear of compassion itself. These difficulties stem from deeply ingrained patterns of negative self‐evaluation, shame, difficulties with cognitive empathy and fear of compassion, all of which contribute to emotional distress and interfere with efforts to develop self‐kindness (Jorgensen and Boye 2024; Naismith et al. 2019). These barriers are often reinforced by social isolation, rejection sensitivity and ineffective emotion regulation strategies, which further limit an individual's ability to extend or receive compassion (Salgó et al. 2021; Scheibner et al. 2018; Valikhani et al. 2020).

One of the most pervasive obstacles is self‐judgment and negative self‐perception, which often lead individuals to over‐identify with distressing emotions and adopt a harsh, self‐critical inner dialogue (Carreiras et al. 2022; Rüsch et al. 2007). Negative self‐judgment involves harsh self‐evaluation, including feelings of worthlessness, failure and self‐stigma, which are particularly prevalent in individuals with borderline personality disorder (Rüsch et al. 2007; Zanarini et al. 2006). For many, this manifests as chronic self‐loathing, difficulties in self‐validation and pervasive self‐doubt, reinforcing a cycle of internal criticism that inhibits compassionate engagement (Krawitz 2012a, 2012b; Moltu et al. 2023; Southward et al. 2023). These deeply entrenched negative self‐appraisals are frequently linked to feelings of unworthiness and social disconnection, making it difficult for individuals to extend the same kindness to themselves that they might offer to others. In addition, low mindfulness and emotion regulation difficulties have been associated with greater struggles in developing a compassionate self‐view, as these factors impair the ability to step back from distressing thoughts and respond with self‐kindness (Salgó et al. 2021; Scheibner et al. 2018; Valikhani et al. 2020).

For individuals with BPD, along with self‐judgement, shame is also often deep‐seated and pervasive, presenting as self‐hatred, self‐destructive behaviours and a sense of personal deficiency (Jorgensen and Boye 2024). These feelings not only contribute to emotional distress but also lead individuals to reject compassion from others, as they feel undeserving of kindness or support. Over time, shame‐based avoidance of compassion can further contribute to isolation in individuals with BPD, reinforcing their low self‐image, deepening their struggles with self‐compassion and limiting opportunities for healing (Jorgensen and Boye 2024).

In addition to these internal struggles, difficulties with cognitive empathy may further hinder the ability to engage in compassionate interactions. While individuals with BPD often experience heightened emotional sensitivity, challenges in understanding and processing emotions can disrupt the ability to form reciprocal, compassionate relationships (Grzegorzewski et al. 2019). Some individuals may exhibit lower levels of cognitive empathy, meaning they struggle to accurately interpret the emotions of others, which can create relational misunderstandings and social frustration. This difficulty is compounded by externally oriented thinking, which may limit introspection and the capacity to reflect on emotional experiences in a way that fosters self‐compassion (Grzegorzewski et al. 2019). As a result, individuals may find both giving and receiving compassion overwhelming or inaccessible, reinforcing feelings of disconnection and interpersonal strain.

A particularly complex barrier is the fear of compassion, which can manifest as discomfort with both self‐directed and externally received kindness (Naismith et al. 2019). Many individuals with BPD struggle with compassion due to past experiences of conditional, unreliable or even harmful caregiving, leading to mistrust and doubts about the authenticity of compassionate gestures (Naismith et al. 2019; Sommerfeld and Bitton 2020). This fear is often heightened by rejection sensitivity, where perceived inconsistencies in compassion may be interpreted as signals of impending abandonment or insincerity (Sommerfeld and Bitton 2020). Consequently, both self‐compassion practices and supportive relationships can trigger emotional distress rather than relief, reinforcing avoidance of emotional closeness.

These internal barriers have far‐reaching implications for interpersonal relationships, as they influence both self‐perception and social interactions. The fear of compassion has been identified as a major obstacle to conflict resolution and trust‐building, particularly among individuals with conditions co‐occurring with BPD, such as addiction (Gratz et al. 2022). Emotional reactivity and withdrawal, key features of BPD, often complicate relational dynamics, making it challenging to seek or accept support in times of distress (American Psychiatric Association 2022; Gratz et al. 2022; Grzegorzewski et al. 2019). Additionally, some individuals with BPD may avoid offering compassion to others, especially when emotional closeness feels threatening or overwhelming (Gratz et al. 2022; Grzegorzewski et al. 2019). This defensive withdrawal serves as a coping mechanism to protect against perceived vulnerability or rejection, yet it can also exacerbate social isolation and interpersonal instability (Gratz et al. 2022).

##### 3.2.2.4 Integrating Compassion Into BPD Therapies

Although this review primarily focuses on identifying barriers to compassionate acts among individuals with BPD, it is important to consider how integrating compassion into therapeutic approaches could help address some of these barriers. Some therapeutic methods have shown promise when compassion is incorporated, yet significant challenges remain (Feliu‐Soler et al. 2017; Krawitz 2012a, 2012b; Naismith et al. 2019; Scheibner et al. 2018; Soler et al. 2022).

One such intervention is Mentalisation‐Based Treatment (MBT), an evidence‐based therapy for BPD that targets underlying difficulties in self‐perception, emotional regulation and interpersonal functioning (Bateman and Fonagy 2004). MBT focuses on enhancing an individual's ability to reflect on their own and others' mental states, helping to reduce self‐stigma, increase self‐compassion and improve relational stability (Luyten et al. 2020). Given that fears of compassion, self‐isolation and difficulties forming compassionate relationships with others are a key factors in limiting compassionate engagement (Fagan et al. 2022; Gratz et al. 2022; Grzegorzewski et al. 2019; Jorgensen and Boye 2024), MBT provides a structured framework for tackling these challenges while promoting healthier interpersonal connections.

However, while interventions such as MBT and Dialectical Behaviour Therapy (DBT) offer structured approaches to emotion regulation and self‐reflection, the integration of compassion‐focused elements remains a complex process. Some therapeutic methods have demonstrated promise when incorporating compassion, yet significant challenges remain (Feliu‐Soler et al. 2017; Krawitz 2012a, 2012b; Naismith et al. 2019; Scheibner et al. 2018; Soler et al. 2022). A key difficulty is that self‐compassion interventions may inadvertently trigger distress, self‐criticism or feelings of invalidation, particularly among individuals with deep‐seated self‐hatred (Donald et al. 2019; Wilner et al. 2024). In some cases, group‐based interventions may intensify feelings of alienation, as comparing oneself to others can reinforce a sense of failure or defeat rather than provide support (Donald et al. 2019). These challenges underscore the necessity of trauma‐informed, highly individualised approaches, ensuring that self‐compassion practices feel accessible and beneficial rather than overwhelming (Wilner et al. 2024).

Despite these complexities, compassion‐based interventions remain an important component of therapeutic approaches for BPD, particularly when integrated into structured treatments. Loving‐kindness and compassion meditation (LKM/CM), for instance, has been associated with improvements in mindfulness, emotional regulation and self‐kindness, though it may not be suitable for all individuals, particularly those experiencing high‐risk behaviours, as the intense emotions elicited can be difficult to regulate (Feliu‐Soler et al. 2017).

## 4 Discussion

This critical review aimed to explore and identify the barriers to compassionate acts for individuals with BPD, focusing on the challenges that hinder their ability to engage in self‐compassion, receive compassion from others and show compassion towards others. Through the analysis of 29 studies, the review highlighted that while engaging in compassion significantly reduces BPD symptoms—such as emotional dysregulation, self‐hatred and interpersonal difficulties—there are substantial barriers to compassionate engagement. These barriers primarily stem from ACEs, stigma and internal challenges, including fear of compassion, shame and low levels of cognitive empathy.

### 4.1 Conceptual Framework

**FIGURE 4:**
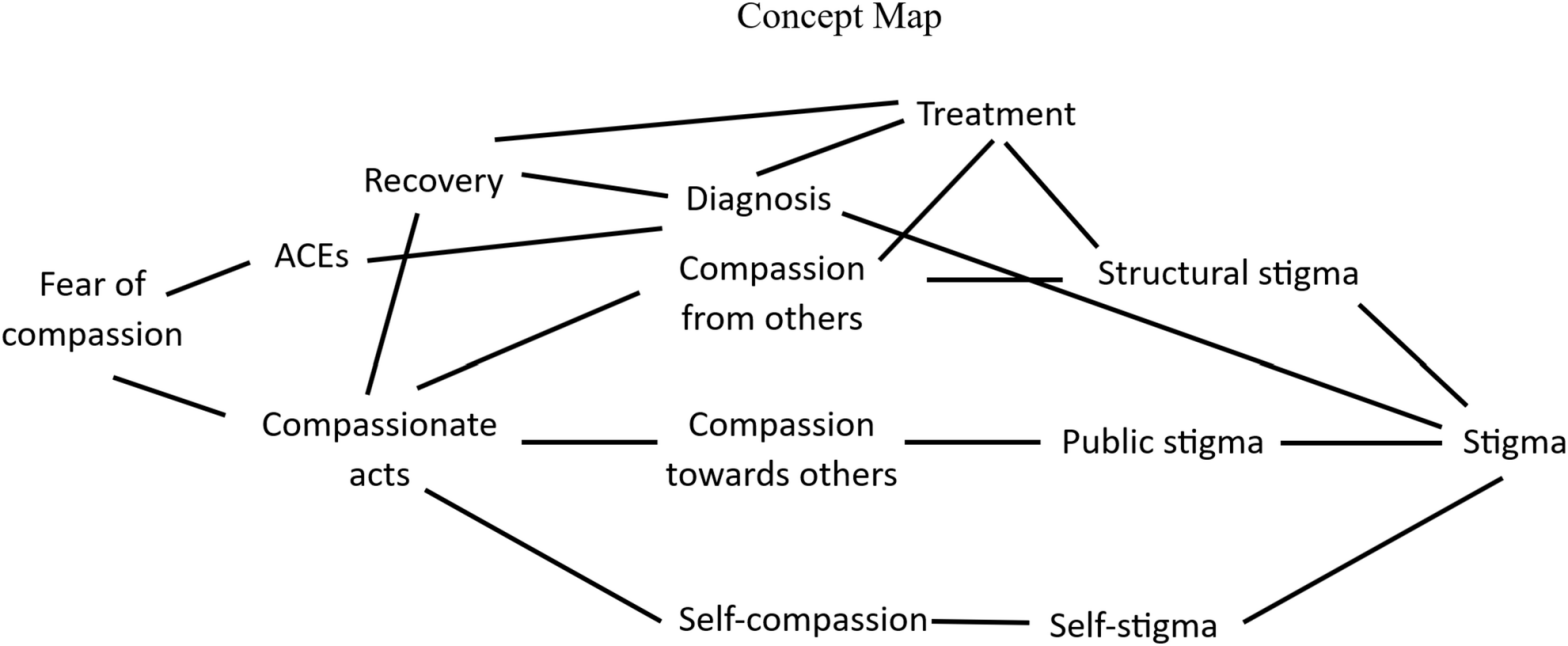
Concept map illustrating interconnected factors influencing compassionate acts among individuals with BPD. Key themes include adverse childhood experiences (ACEs), fear of compassion, self‐stigma, public stigma, structural stigma and various types of compassion (e.g., from others, towards others and self‐compassion). Arrows indicate conceptual relationships between constructs.

This review identifies multiple interrelated barriers to compassionate engagement among individuals with BPD. These include early‐life adversity in the form of ACEs, social exclusion and stigma, and internal psychological struggles such as self‐judgment, shame and fear of compassion (Figure 4). While compassion‐focused interventions offer promise, their efficacy is often hindered by unaddressed structural and relational challenges. This conceptual framework visually maps these barriers in relation to one another, demonstrating how societal and interpersonal factors interact with intrapersonal difficulties to limit compassionate engagement. The framework also highlights how stigma and structural discrimination (i.e., sanism) may exacerbate existing psychological vulnerabilities, reinforcing cycles of self‐criticism and social withdrawal. By integrating both individual‐level and systemic factors, this model illustrates the complexity of barriers to compassion in BPD and highlights the necessity of multi‐level interventions that address personal, clinical, and broader policy‐driven obstacles to recovery.

The conceptual map also highlights the systemic influences that shape an individual's ability to engage in compassion. A BPD diagnosis can be a double‐edged sword, facilitating access to treatment while simultaneously increasing vulnerability to stigma within healthcare settings. Structural stigma, particularly within mental health services, may limit access to compassionate care and reinforce cycles of rejection and self‐stigma. These factors collectively influence recovery outcomes, demonstrating the importance of both individual and systemic interventions. Addressing these barriers requires a multilevel approach that not only fosters self‐compassion and interpersonal trust but also challenges institutional biases and systemic discrimination. By synthesising these relationships, this framework underscores the need for integrated, trauma‐informed approaches that acknowledge both the personal and structural challenges individuals with BPD face in developing compassionate engagement.

A significant barrier to compassionate engagement in BPD arises from structural discrimination within mental health services, which extends beyond interpersonal prejudice to systemic exclusionary practices (Perlin 1993). While stigma is commonly used to describe negative societal and clinical attitudes towards individuals with BPD, it primarily captures individual biases and public misconceptions. However, stigma alone does not fully explain the entrenched discrimination faced by individuals with BPD within institutional settings. To address this limitation, the concept of sanism has been introduced, referring specifically to systemic and structural discrimination against individuals with mental health conditions (Lavallee and Gagné‐Julien 2024). Unlike stigma, which often frames negative attitudes as a problem of perception, sanism highlights how mental health policies, clinical practices and service structures actively exclude and marginalise individuals with BPD.

One of the most pervasive consequences of sanism is the mischaracterisation of BPD within psychiatric frameworks, where the diagnosis is often viewed as too complex, untreatable, or inherently resistant to care. This perception has led to unequal access to services, rejection from treatment and inadequate therapeutic responses, as some clinicians hold stigmatising assumptions about individuals with BPD and their capacity for recovery (Ring and Lawn 2019). These exclusionary practices are not simply the result of personal prejudice among clinicians but are reinforced by broader institutional norms that pathologise BPD, shaping treatment availability, funding priorities and professional training standards (Klein et al. 2022). As a result, individuals with BPD may struggle to access compassionate care, reinforcing cycles of self‐stigma and social withdrawal. Tackling these systemic issues requires institutional reforms that move beyond stigma reduction efforts to prioritise trauma‐informed care, clinician education and service models that actively promote compassionate engagement.

Sanism is also evident in the medicalisation of distress, where individuals with BPD are pathologised rather than understood within the context of trauma and adversity (Lavallee and Gagné‐Julien 2024). This framing prioritises pharmacological interventions at the expense of holistic, person‐centred approaches, such as peer support and relational therapeutic models. While stigma reduction campaigns often aim to change public attitudes, they rarely address these deeper, structural forms of discrimination that impact service provision. By focusing on sanism, a more comprehensive understanding of the barriers to compassionate engagement can be developed—one that acknowledges the interplay between societal attitudes, clinical practices and systemic exclusion. Addressing sanism, therefore, requires policy reform, changes in clinical training, and a re‐evaluation of service structures to ensure that individuals with BPD are not only included but actively supported within mental health care (Beresford et al. 2010). Without confronting these structural barriers, interventions designed to foster compassionate engagement may remain superficial and ineffective. Future research should examine how sanism manifests in clinical practice and explore strategies to dismantle institutional biases that continue to hinder recovery and compassionate care for individuals with BPD.

Although compassion‐focused interventions aim to enhance self‐compassion and reduce self‐criticism, their effectiveness can also be limited by the internal barriers stemming from ACEs. Individuals with BPD may perceive these interventions as invalidating their traumatic experiences, leading to negative reactions and disengagement. This disconnect can result in feelings of defeat and alienation, further exacerbating self‐stigma and reducing self‐compassion. Additionally, the fear of compassion and the presence of self‐hatred complicate the implementation of these interventions, making it challenging for individuals to benefit fully from them.

ACEs are also a significant barrier to engagement in compassionate acts for individuals with BPD. Childhood trauma, including abuse, neglect and domestic violence, is strongly associated with the development of BPD and influences the severity of symptoms. These adverse experiences can lead to negative self‐perceptions and emotional dysregulation, creating substantial internal barriers to engaging in compassionate acts. Self‐compassion, while a promising tool for BPD recovery, is often hindered by these internal barriers, resulting in difficulties in emotional regulation and self‐esteem.

Being unable to access effective treatments due to internal and external barriers can lead to increased self‐stigma. Individuals with BPD may internalise the negative attitudes and judgments they encounter from clinicians, or which occur as a result of unsuitable interventions, resulting in diminished self‐compassion and a perpetuation of negative self‐perceptions. This cyclical pattern of self‐stigma and reduced self‐compassion further hinders recovery and compassionate engagement.

Despite these challenges, individuals with BPD often display genuine compassion towards others, shaped by their own life experiences. However, their ability to engage in compassionate relationships can be hindered by internal barriers and fear of vulnerability. Promoting comfort with compassionate interactions and reducing fears of compassionate engagement are essential for improving interpersonal relationships and enhancing recovery.

### 4.2 Conceptual Critique

The literature on compassionate engagement in BPD often emphasises individual responsibility for behaviour change, overlooking the broader societal context in which negative self‐perceptions develop. ACEs stigma and social rejection significantly contribute to these internal barriers, highlighting the need to examine these social origins more comprehensively. Addressing these sociocultural factors—such as structural discrimination, stigma within healthcare and the exclusion of individuals with BPD from compassionate discourse—is crucial for designing more effective, holistic interventions.

While many studies in this review addressed stigma, none focused specifically on sanism. Stigma, however, may not fully capture the systemic discrimination that shapes negative experiences for individuals with BPD. Incorporating sanism into research could provide a more nuanced understanding of these challenges, leading to more targeted strategies for fostering compassionate engagement. The distinction between general stigma and sanism underscores the need for structural interventions that move beyond individual behaviour change and instead address systemic discrimination faced by individuals with BPD.

Although compassion‐focused interventions have been shown to improve self‐compassion and reduce self‐stigma, the literature often fails to consider broader structural and societal barriers that impact the effectiveness of these interventions. Addressing systemic barriers, including clinician biases, institutional exclusion and public misconceptions, is crucial for fostering compassionate engagement both within and outside of therapeutic settings. Shifting the focus beyond individual responsibility and towards systemic change will be key in developing effective interventions that support recovery.

Therefore, while integrating compassion into BPD therapies is beneficial, it is insufficient on its own. To fully enhance therapeutic outcomes, these efforts must be accompanied by structural changes that address the factors perpetuating stigma, discrimination and exclusion. Creating more compassionate environments, both within clinical settings and society at large, will significantly improve the effectiveness of these interventions, ultimately facilitating more meaningful compassionate engagement for individuals with BPD.

### 4.3 Methodological Limitations and Future Research Directions

The studies reviewed predominantly employed cross‐sectional designs, which provide insight into associations at specific points in time but limit the ability to infer causation or track long‐term changes. Future research should prioritise longitudinal studies to examine the sustained impact of compassionate engagement on BPD symptoms and overall well‐being.

A notable gender imbalance exists within the literature, with most studies focusing on predominantly female participants. Given growing evidence that BPD is diagnosed at similar rates in men and women, this overrepresentation may limit the generalisability of findings (Bozzatello et al. 2024). Future studies should prioritise greater gender diversity to explore differences in self‐compassion and stigma experiences among men, women and gender‐diverse individuals (Qian et al. 2022). Additionally, age variation across studies suggests a need to consider how self‐compassion and barriers to compassionate engagement evolve across different life stages (Bratt and Fagerström 2020).

A major gap in the literature is the lack of participatory research involving individuals with lived experience of BPD. Many of the barriers to compassionate engagement relate to interactions with mental health services, making first‐hand perspectives crucial for developing effective interventions. Future research should actively involve individuals with BPD in research design to ensure interventions are practical, relevant and beneficial.

There is also a pressing need for randomised controlled trials (RCTs) to evaluate the effectiveness of compassion‐focused interventions in BPD treatment. While some evidence suggests that CFT, DBT and MBT may enhance self‐compassion and emotional regulation, existing findings remain limited to small‐scale studies. Future research should prioritise large‐scale RCTs to assess how these interventions influence self‐stigma, compassionate engagement and long‐term recovery outcomes.

Additionally, many of the reviewed studies relied on self‐reported data, which, while valuable for capturing subjective experiences, is prone to biases such as social desirability and emotional fluctuation. The limited use of qualitative methods further restricts the depth of understanding, highlighting the need for mixed‐method approaches. Studies such as Koivisto et al. (2022), which combine qualitative and quantitative methods, provide richer insights into barriers to compassionate engagement. The use of Ecological Momentary Assessment (EMA) or Experience Sampling Methods (ESM) could further enhance real‐time insights into fluctuations in self‐compassion and interpersonal engagement, offering a more immediate and nuanced understanding of these experiences (Trull and Ebner‐Priemer 2009).

Although the majority of studies included in this review were conducted in WEIRD countries (e.g., the Unites States, Australia and several European nations), a smaller number of studies originated from non‐WEIRD contexts such as Iran and Singapore. While this limited geographic diversity provides some insight into cross‐cultural differences, the predominance of WEIRD‐based research means that the findings may not fully generalize to non‐WEIRD populations. Future studies should prioritize underrepresented regions to enhance the applicability of research on compassionate engagement in BPD across diverse cultural and socio‐economic contexts.

By integrating participatory research, longitudinal designs, rigorous RCTs and qualitative methodologies, future studies could advance our understanding of the barriers to compassionate engagement in BPD, leading to more effective therapeutic approaches and policy changes.

### 4.4 Implications for Clinical Practice and Society

For individuals with BPD, understanding barriers to compassionate engagement provides clarity on the challenges associated with self‐compassion, receiving compassion from others, and engaging in compassionate acts. Recognising that these barriers often stem from ACEs, stigma and internal struggles such as self‐criticism and shame may help individuals seek more targeted mental health support. Awareness of structural barriers also helps validate these experiences, reducing self‐blame and fostering more realistic self‐expectations.

For service providers, this review highlights the importance of training clinicians to recognise and reduce stigma when working with individuals with BPD. Negative clinician attitudes and prejudicial assumptions about BPD can exacerbate self‐stigma and impede therapeutic engagement, making it crucial to promote non‐stigmatising, trauma‐informed approaches. Reducing sanism in mental healthcare services and implementing interventions tailored to the specific challenges faced by individuals with BPD could improve engagement in therapy and enhance recovery outcomes. Additionally, policymakers must prioritise systemic changes that address structural discrimination within mental health services and promote a culture of compassion in clinical care.

At a broader societal level, fostering greater public awareness, empathy and inclusive mental health policies is essential for reducing the stigma and isolation experienced by individuals with BPD. Educational initiatives that promote early learning around emotional intelligence, mental health literacy and compassionate communication could challenge misconceptions about BPD and foster more supportive social environments. Community‐based interventions that prioritise social connection and mutual aid could further empower individuals with BPD by creating environments where compassionate engagement is actively encouraged and modelled.

A dual approach—integrating systemic change with evidence‐based therapeutic strategies—will be critical in reducing stigma, improving mental health outcomes and ensuring that compassionate engagement is both accessible and sustainable.

## 5 Conclusion

This critical review has explored the significant barriers to compassionate acts among individuals with BPD including ACEs, stigma and internal challenges such as fear of compassion and self‐criticism. These barriers not only hinder compassionate engagement but also exacerbate the symptoms of BPD, such as emotional dysregulation, self‐hatred and interpersonal difficulties.

Understanding these obstacles is crucial for developing targeted, trauma‐informed interventions that can enhance compassionate engagement and support recovery in individuals with BPD. The review emphasises the importance of integrating compassion into therapeutic approaches while also advocating for broader systemic changes. These include reducing sanism within mental health care, promoting non‐stigmatising communication among clinicians and fostering a societal culture that values compassion and empathy.

Additionally, there is a need for more comprehensive research, particularly studies that include longitudinal designs, participatory approaches involving individuals with lived experiences, and mixed‐methods research. Such studies are essential to deepen the understanding of the complex relationships between self‐compassion, ACEs, stigma and BPD symptoms.

Addressing both individual and systemic barriers can create a more supportive environment that facilitates compassionate engagement and improves the overall well‐being of individuals with BPD. This holistic approach enhances the effectiveness of therapeutic interventions and contributes to a broader societal shift toward empathy and inclusivity, ultimately supporting the recovery journey of those affected by BPD.

## Ethics Statement

Ethical approval was not required for publication of this a Critical Review.

## Conflicts of Interest

The authors declare no conflicts of interest.

## Data Availability

Data sharing is not applicable to this article as no datasets were generated or analysed during the current study.
